# Glucocorticoid Abnormalities in Female Rats Exposed to a Predator-Based Psychosocial Stress Model of PTSD

**DOI:** 10.3389/fnbeh.2021.675206

**Published:** 2021-06-18

**Authors:** Phillip R. Zoladz, Colin R. Del Valle, Eva A. Smith, Cassandra S. Goodman, Jordan L. Dodson, Kara M. Elmouhawesse, Charis D. Kasler, Boyd R. Rorabaugh

**Affiliations:** ^1^Psychology Program, The School of Health and Behavioral Sciences, Ohio Northern University, Ada, Ohio, OH, United States; ^2^Department of Pharmaceutical Sciences, Marshall University School of Pharmacy, Huntington, WV, United States

**Keywords:** corticosterone, females, PTSD, animal model, HPA axis, anxiety

## Abstract

People with post-traumatic stress disorder (PTSD) exhibit heightened anxiety and enhanced negative feedback of the hypothalamus-pituitary-adrenal (HPA) axis. We previously reported that male rats exposed to a predator-based psychosocial stress model of PTSD exhibited comparable changes in anxiety-like behavior and HPA axis activity, including lower baseline levels of corticosterone and a greater suppression of corticosterone after dexamethasone administration. Here, we assessed whether we would observe similar effects in female rats exposed to this model. Adult female Sprague-Dawley rats were exposed to a cat on two occasions (separated by 10 days), in combination with chronic social instability. Three weeks after the second cat exposure, we assessed anxiety-like behavior on an elevated plus maze (EPM) and collected blood samples from rats in the absence or presence of dexamethasone to quantify serum corticosterone levels. Although stressed females did not display heightened anxiety on the EPM, they exhibited significantly lower overall corticosterone levels and a greater suppression of corticosterone after dexamethasone administration. The observation of significantly lower overall corticosterone levels in stressed females was replicated in a separate, independent experiment. These findings suggest that the predator-based psychosocial stress model of PTSD may be useful for studying mechanisms that underlie changes in HPA axis function in females exposed to trauma.

## Introduction

Post-traumatic stress disorder (PTSD) is a debilitating psychiatric condition characterized by heightened anxiety, hyperarousal, intrusive memories, cognitive impairments, and several deleterious physiological symptoms (Zoladz and Diamond, 2013). One physiological system extensively studied in PTSD patients is the hypothalamus-pituitary-adrenal (HPA) axis. Although findings have been mixed (Meewisse et al., 2007; Speer et al., 2019), research has generally shown that people with PTSD display abnormally low levels of cortisol, a greater suppression of cortisol following dexamethasone administration, and increased glucocorticoid receptor sensitivity (Lehrner et al., 2016). These HPA axis alterations might exist prior to PTSD onset and confer increased risk for the disorder or reflect the pathophysiology of trauma- and/or PTSD-related symptoms.

Our laboratory utilizes a predator-based psychosocial stress model of PTSD that results in numerous physiological and behavioral changes in rats that are comparable to those observed in PTSD patients. The model was explicitly designed to incorporate features that are known to be associated with increased susceptibility for PTSD in traumatized individuals (Zoladz et al., 2015). It consists of a 31-day paradigm, during which rats are exposed to an adult, female cat (i.e., predator stress) on two occasions (separated by 10 days) and experience chronic social instability by having their cage mates changed daily (Zoladz et al., 2008). The two cat exposures are meant to mimic the re-experiencing symptoms that typify PTSD, and the social instability component provides a chronic mild stressor meant to mirror the inconsistent social environment experienced by individuals with PTSD. Because the model involves repeated stress, it may be most applicable to understanding PTSD that results from multiple trauma exposures (e.g., combat, domestic abuse) (Zoladz et al., 2008). Previous work has shown that male rats exposed to this model exhibit increased anxiety on the elevated plus maze, a robust memory of the two cat exposures, impaired cognitive function, an exaggerated startle response, increased heart rate and blood pressure, greater myocardial sensitivity to ischemic injury, reduced levels of serotonin, higher levels of norepinephrine, increased measures of inflammation and oxidative stress, and elevated methylation of hippocampal *Bdnf* DNA (Zoladz et al., 2008, 2012, 2013, 2015; Roth et al., 2011; Wilson et al., 2013, 2014a,b,c; Rorabaugh et al., 2015). We have also reported that males exposed to this model demonstrate lower baseline levels of corticosterone and a greater suppression of corticosterone following treatment with dexamethasone (Zoladz et al., 2012), comparable to the findings of some other animal models (Kohda et al., 2007; Pooley et al., 2018). However, few animal models of PTSD have incorporated females; even fewer have examined HPA axis function in females, the findings of which have been inconsistent (Louvart et al., 2005, 2006; Pooley et al., 2018). Recently, we extended upon the predator-based psychosocial stress model of PTSD to show that females exposed to the stress paradigm exhibited PTSD-like increases in anxiety that were not dependent on estrous stage or ovarian hormones (Zoladz et al., 2019). In the present study, we examined whether this model of PTSD would produce the same PTSD-like glucocorticoid abnormalities in females that were previously observed in males.

## Materials and Methods

### Subjects

Adult female Sprague-Dawley rats (~10 weeks of age) from an established breeding colony at Ohio Northern University were housed on a 12-h light/dark schedule (lights on at 0700 h) in standard Plexiglas cages (two per cage) with free access to food and water. All procedures were approved by the Institutional Animal Care and Use Committee at Ohio Northern University and followed those recommended by the Guide for the Care and Use of Laboratory Animals provided by the National Institutes of Health.

### Chronic Stress Procedure

We have described the chronic stress procedure at length in previous publications (Zoladz et al., 2008, 2012, 2013, 2015, 2019; Roth et al., 2011; Rorabaugh et al., 2015). In brief, rats were assigned to “stress” or “no stress” groups. On Day 1, rats in the stress groups were immobilized in plastic DecapiCones (Braintree Scientific; Braintree, MA) and then placed in a perforated wedge-shaped Plexiglas enclosure (Braintree Scientific; Braintree, MA; 20 × 20 × 8 cm). This enclosure was then taken to a cat housing room and placed in a metal cage (61 × 53 × 51 cm) with an adult female cat. One hour later, the rats were returned to their home cages. Rats in the no stress groups remained in their home cages during the 1-h stress period. Rats in the stress groups were given two cat exposures, which were separated by 10 days. The first cat exposure took place during the light cycle (between 0800 and 1200 h), and the second cat exposure took place during the dark cycle (between 1900 and 2100 h).

Beginning on the day of the first cat exposure (Day 1), rats in the stress groups were exposed to chronic social instability until the day (Day 32) of blood sampling (Experiments 1 and 2) or behavioral testing (Experiment 3). Rats in the stress groups were housed two per cage, but every day, their cage mates were changed. Rats in the no stress groups were housed with the same cage mates throughout the experiment and handled daily.

On Day 31 (i.e., 20 days after the second cat exposure), the hind legs of all rats were shaved to allow access to their saphenous veins. The rats were then taken back to the housing room and left undisturbed for the remainder of the day.

Rats were weighed on the days of the first and second cat exposures, as well as on Day 32. Growth rates during the stress paradigm [g/day = (weight on Day 32 – weight on the day of the first cat exposure)/31] were calculated for statistical analysis.

### Behavioral Testing

In Experiment 3, rats were tested on the elevated plus maze (EPM) to assess anxiety-like behavior. A red light (25-watt, 15 lumens) that was mounted above the EPM was used to provide low ambient lighting during testing, similar to previous research (e.g., Savic et al., 2004; Violle et al., 2009; Morena et al., 2016). The rats were placed on the EPM for 5 min, and their behavior was videotaped with a JVC hard disk camera hanging above the EPM and scored offline by two separate investigators who were blind to the experimental conditions of the animals. The investigators scored the amount of time rats spent in the open arms of the EPM, as well as the number of entries rats made into the open and closed arms (a measure of overall locomotor activity).

### Experiment 1: Glucocorticoid Levels in the Absence of Dexamethasone

All blood sampling methodology was identical to that which was employed in our previous work in males (Zoladz et al., 2012). Three weeks after the second cat exposure (Day 32), beginning at 1700 h, stressed (*n* = 19) and non-stressed (*n* = 15) rats were taken individually to a procedure room with normal overhead lighting for blood sampling. Because serum corticosterone levels are quite dynamic at this time of day in rodents, the timing of blood sampling was carefully counterbalanced across both groups. Petroleum jelly was applied to each rat's hind leg, and the saphenous vein of each rat was punctured with a sterile, 27-gauge syringe needle. A 0.2 cc sample of blood was then collected from each rat within 2 min of being removed from the housing room (0 min time point). All blood sampling commenced prior to the onset of the dark cycle in the housing room (i.e., prior to 1900 h). After obtaining this sample, the rats were immobilized in plastic DecapiCones for 20 min. Then, another 0.2 cc sample was collected via saphenous vein venipuncture (20 min time point). After the second sample was collected, the rats were returned to their home cages and taken to a holding room with normal overhead lighting. One hour later, a final sample was collected from all rats (80 min time point) in the procedure room described above. All three blood samples were collected from rats prior to 2000 h.

### Experiment 2: Dexamethasone Suppression Test

Three weeks after the second cat exposure (Day 32), between 1100 and 1400 h, stressed (10 μg/kg: *n* = 15; 25 μg/kg: *n* = 15; vehicle: *n* = 10) and non-stressed (10 μg/kg: *n* = 14; 25 μg/kg: *n* = 15; vehicle: *n* = 10) rats were administered subcutaneous injections of dexamethasone (10 μg/kg, 25 μg/kg) or vehicle at a volume of 1 ml/kg. Previous work indicated that these doses produce a modest suppression of corticosterone levels in rats (Lurie et al., 1989), and in males exposed to the present model of PTSD, these doses were most effective in revealing stress-induced HPA axis alterations (Zoladz et al., 2012). Dexamethasone was dissolved in a vehicle consisting of sodium sulfite (1 mg/ml) and sodium citrate (19.4 mg/ml), which were both dissolved in distilled water. Six hours following the injections (Lurie et al., 1989; Zoladz et al., 2012), blood sampling commenced, as described in Experiment 1. As in Experiment 1, the timing of blood sampling was carefully counterbalanced across all groups.

### Experiment 3: Anxiety-Like Behavior and Replication of Glucocorticoid Abnormalities

Experiment 3 was conducted to assess anxiety-like behavior in female rats exposed to chronic stress and to replicate corticosterone findings from Experiments 1 and 2. Specifically, we aimed to replicate the finding of lower corticosterone levels in non-injected, stressed females (Experiment 1), an effect that was eliminated by injecting such rats with vehicle 6 h prior to blood sampling (Experiment 2).

Three weeks after the second cat exposure (Day 32), all rats were tested on the EPM for 5 min. The next day, between 1100 and 1400 h, stressed and non-stressed rats were administered subcutaneous injections of the vehicle utilized in Experiment 2 (stress: *n* = 10; no stress: *n* = 10) or were left undisturbed in their home cages (stress: *n* = 10; no stress: *n* = 10). Six hours later, blood sampling commenced, as described above. As in Experiments 1 and 2, the timing of blood sampling was carefully counterbalanced across all groups.

**Figure 1:**
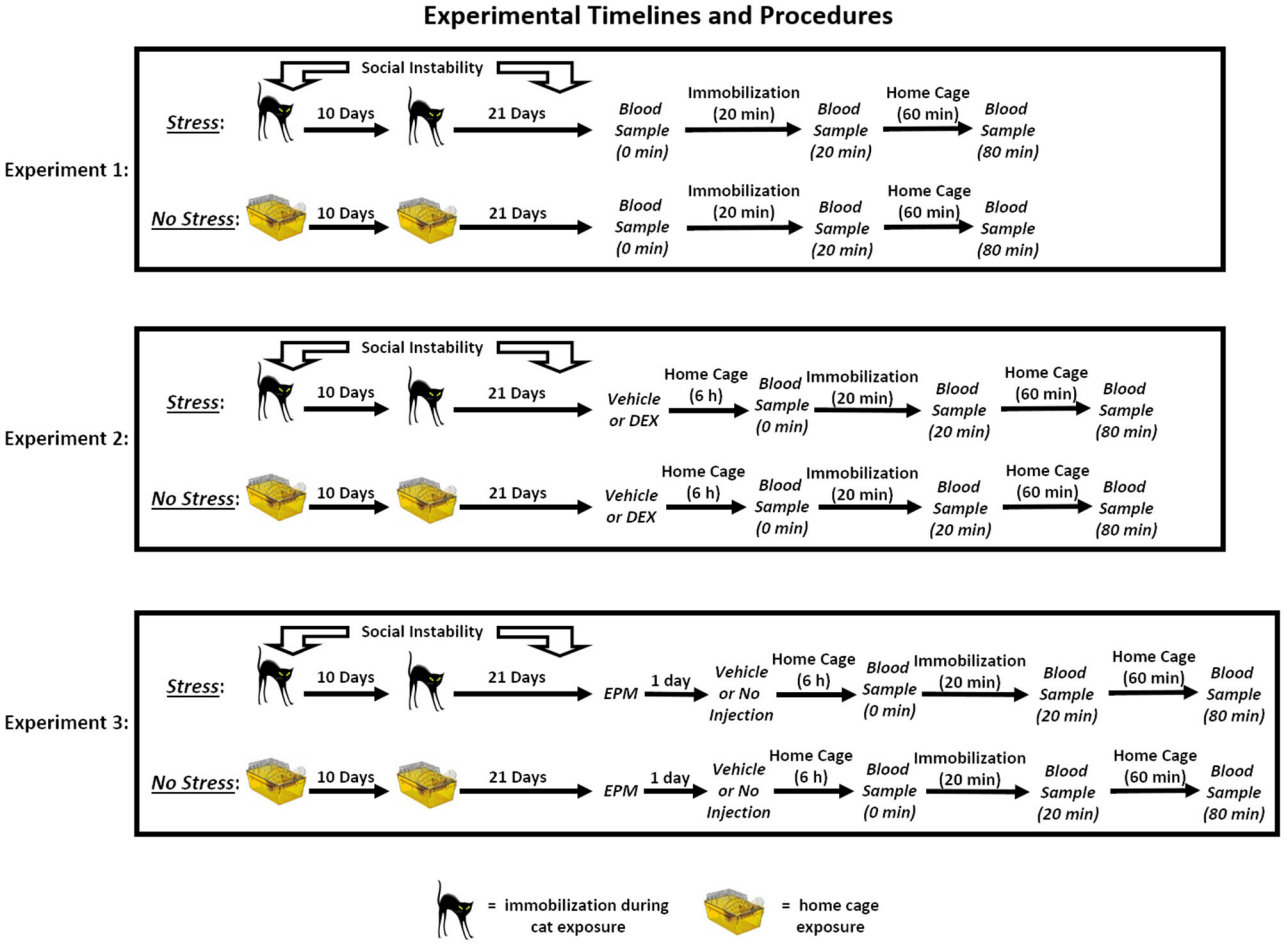
Experimental timelines and procedures. In Experiment 1, rats in the stress group were exposed to a 31-day chronic stress paradigm that consisted of two cat exposures and daily social instability. Stressed rats were immobilized and exposed to an adult, female cat for 1 h on Days 1 (during the light cycle) and 11 (during the dark cycle), and throughout the 31-day paradigm, the cage mates of stressed rats were randomly changed on a daily basis. Rats in the no stress group remained in their home cages on Days 1 and 11 and remained with the same cage mate throughout the paradigm. On Day 32, three blood samples were collected from the rats. The first sample was collected within 2 min of removing the rats from their home cages (0 min), and then the rats were immobilized for 20 min. Following the acute immobilization, a second blood sample was collected to assess acute stress-induced changes in corticosterone levels (20 min). The rats were then placed in their home cages for 1 h, after which a final blood sample was collected (80 min). In Experiment 2, the rats underwent the stress or no stress manipulations described above and then, on Day 32, were subcutaneously injected with 10 or 25 μg/kg dexamethasone (DEX) or vehicle 6 h prior to undergoing blood sampling as described for Experiment 1. In Experiment 3, the rats underwent the stress or no stress manipulations described above and then, on Day 32, were tested on the elevated plus maze (EPM) for 5 min. On Day 33, the rats were subcutaneously injected with vehicle or were not injected 6 h prior to undergoing blood sampling as described for Experiment 1.

The timeline for each of the three experiments can be found in Figure 1.

### Corticosterone Assays

Once the blood samples had clotted at room temperature, they were centrifuged (6000 rpm for 8 min, average relative centrifugal force = 1,200 × g), and the serum was extracted and stored at −80°C until assayed for corticosterone with an enzyme immunoassay kit (Enzo Life Sciences; Farmingdale, NY) according to the manufacturer's instructions. The collected serum was diluted 40-fold using assay buffer that was provided by the manufacturer. Corticosterone was displaced from binding proteins with a steroid displacement reagent; thus, the assay measured total serum corticosterone. The sensitivity of the assay was 0.0027 μg/dl, and the intra- and inter-assay coefficients of variation were 2.88 and 12.82%, respectively.

### Statistical Analyses

Independent samples *t*-tests were used to compare the growth rates and EPM behavior (Experiment 3) of stressed and non-stressed rats. A 2-way mixed-model ANOVA was used to analyze the corticosterone data in Experiment 1, with stress serving as the between-subjects factor and blood sample time point serving as the within-subjects factor. A 3-way mixed-model ANOVA was used to analyze the corticosterone data in Experiments 2 and 3, with stress and drug/injection serving as the between-subjects factors and blood sample time point serving as the within-subjects factor. Alpha was set at 0.05, and Bonferroni-corrected planned comparisons or *post-hoc* tests were employed when indicated. Greenhouse-Geisser corrections were employed if the assumption of sphericity was violated, and this is denoted by reduced numerator degrees of freedom for the *F* statistic.

## Results

### Experiment 1: Glucocorticoid Levels in the Absence of Dexamethasone

#### Growth Rates

Stressed females (*M* = 0.66 g/day, *SEM* = 0.072) gained significantly less weight than non-stressed females (*M* = 1.02 g/day, *SEM* = 0.079), *t*(30) = 3.27, *p* = 0.003.

#### Corticosterone Levels

**Figure 2:**
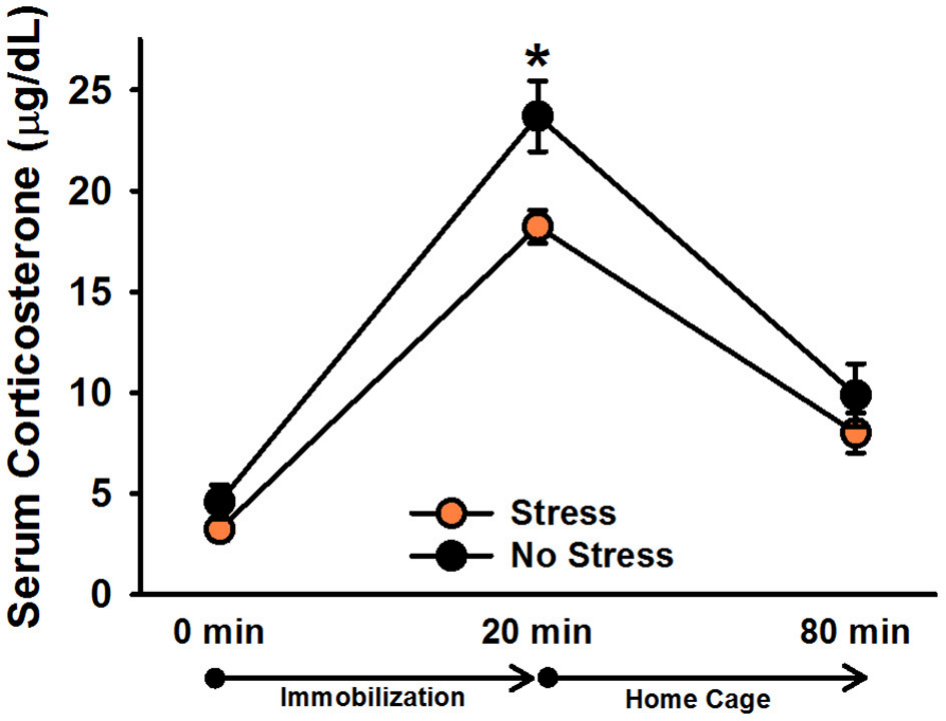
Serum corticosterone levels in non-injected female rats. Stressed females exhibited significantly lower overall corticosterone levels than non-stressed females, which was particularly driven by the difference in corticosterone levels following 20 min of immobilization. Data are presented as means ± SEM. *p < 0.01.

There was a significant effect of time point, [*F*_(2, 60)_ = 154.22, *p* < 0.001]. *Post-hoc* tests indicated that corticosterone levels significantly increased following 20 min of immobilization, *t*(31) = 15.91, corrected *p* < 0.001, and then significantly decreased after rats spent an hour in their home cages, *t*(31) = 12.97, corrected *p* < 0.001 (Figure 2). There was also a significant effect of stress, [*F*_(1, 30)_ = 5.84, *p* = 0.022], indicating that, overall, stressed females exhibited significantly lower corticosterone levels than non-stressed females. Although the Stress × Time Point interaction was not significant, [*F*_(2, 60)_ = 2.51, *p* = 0.09], we performed Bonferroni-corrected comparisons to examine corticosterone levels between stressed and non-stressed females at each time point. These comparisons revealed that stressed females exhibited significantly lower corticosterone levels than non-stressed females following 20 min of immobilization, *t*(30) = 2.79, corrected *p* = 0.009, but not at baseline, *t*(30) = 1.29, corrected *p* = 0.206, or an hour following the termination of immobilization, *t*(30) = 1.10, corrected *p* = 0.280.

### Experiment 2: Dexamethasone Suppression Test

#### Growth Rates

The analysis of growth rates revealed no significant difference between stressed females (*M* = 0.79 g/day, *SEM* = 0.05) and non-stressed females (*M* = 0.80 g/day, *SEM* = 0.06), *t*(73) = 0.32, *p* = 0.97.

#### Corticosterone Levels

**Figure 3:**
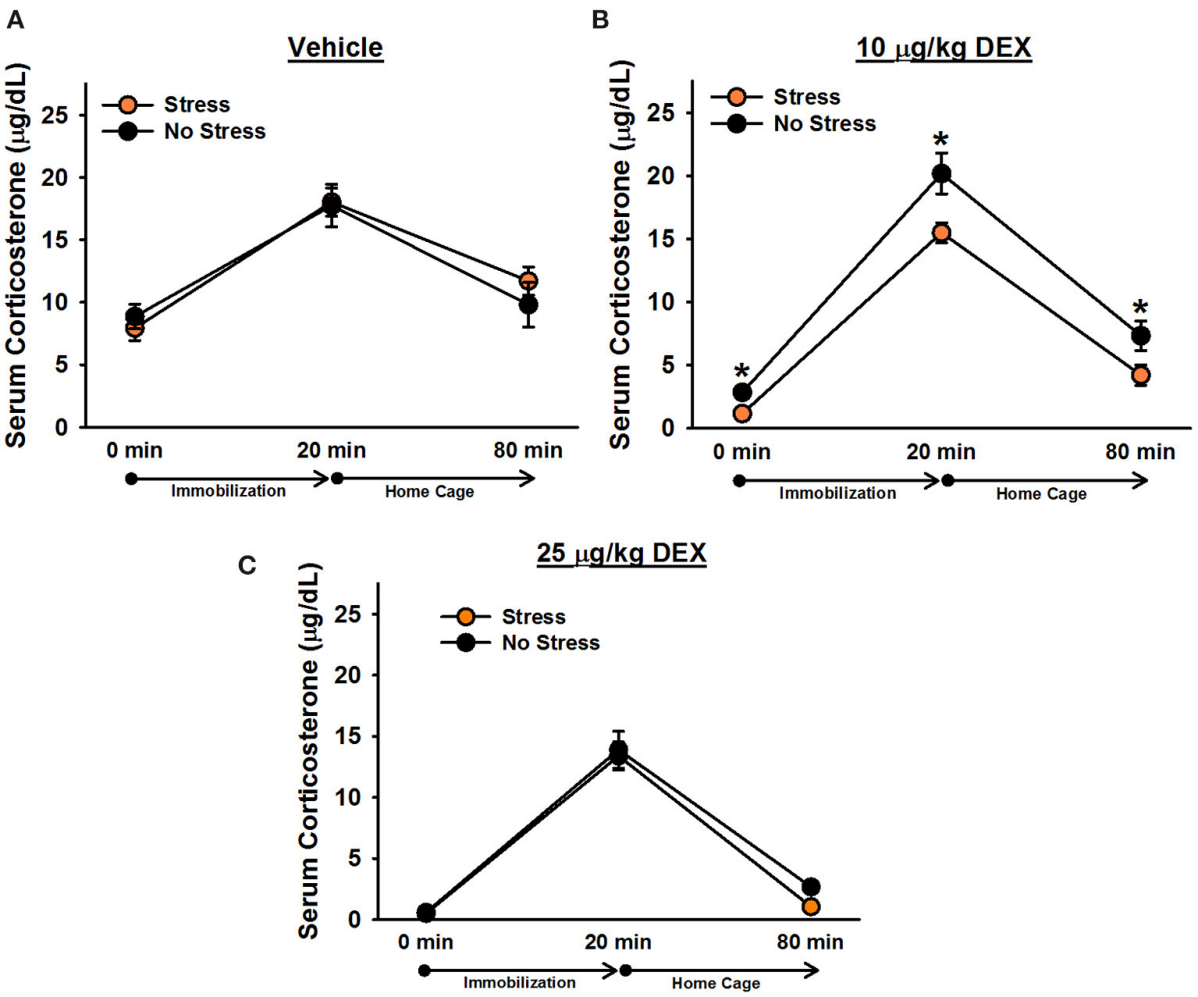
Serum corticosterone levels in female rats administered vehicle **(A)**, 10 μg/kg dexamethasone **(B)**, or 25 μg/kg dexamethasone **(C)**. Stressed females exhibited significantly lower corticosterone levels than non-stressed females, overall, following administration of 10 μg/kg dexamethasone. Planned comparisons on these data revealed that stressed females had significantly lower corticosterone levels than non-stressed females at every time point. Data are presented as means ± SEM. *p < 0.05.

There was a significant effect of time point, [*F*_(1.8, 126)_ = 285.19, *p* < 0.001]. *Post-hoc* tests indicated that corticosterone levels significantly increased following 20 min of immobilization, *t*(68) = 20.39, corrected *p* < 0.001, and then significantly decreased after rats spent an hour in their home cages, *t*(68) = 16.30, corrected *p* < 0.001 (Figure 3). There was also a significant effect of drug, [*F*_(2, 63)_ = 52.49, *p* < 0.001], verifying that dexamethasone significantly reduced corticosterone levels. *Post-hoc* tests revealed that rats administered 25 μg/kg dexamethasone exhibited significantly lower overall corticosterone levels than rats administered 10 μg/kg dexamethasone, *t*(50) = 5.47, corrected *p* < 0.001, and rats administered 10 μg/kg dexamethasone exhibited significantly lower overall corticosterone levels than rats administered vehicle, *t*(40) = 4.13, corrected *p* < 0.001. The Drug × Time Point interaction was also significant, [*F*(_3.6,126)_ = 5.96, *p* < 0.001]. *Post-hoc* tests indicated that, relative to vehicle, 25 μg/kg dexamethasone significantly blunted rats' corticosterone response to 20 min immobilization stress, *t*(42) = 3.60, corrected *p* = 0.003; however, rats administered 10 μg/kg dexamethasone exhibited immobilization-induced increases in corticosterone levels that were statistically equivalent to those of rats administered vehicle, *t*(40) = 0.02, corrected *p* = 1.000. Similar to Experiment 1, there was a significant effect of stress, [*F*_(1, 63)_ = 4.54, *p* = 0.037], revealing that stressed females displayed significantly lower overall corticosterone levels than non-stressed females. This effect depended on drug condition {Stress × Drug interaction: [*F*_(2, 63)_ = 4.43, *p* = 0.016]}. *Post-hoc* tests showed that stressed females exhibited significantly lower overall corticosterone levels than non-stressed females only when they were administered 10 μg/kg dexamethasone, *t*(23) = 3.98, corrected *p* < 0.001. Although the Stress × Drug × Time Point interaction was not significant, [*F*_(4, 126)_ = 1.28, *p* = 0.28], we performed Bonferroni-corrected comparisons at each time point of blood sampling to examine corticosterone levels between stressed and non-stressed females administered 10 μg/kg dexamethasone. These comparisons revealed that stressed females administered 10 μg/kg dexamethasone exhibited significantly lower corticosterone levels than non-stressed females administered 10 μg/kg dexamethasone at baseline, *t*(23) = 3.84, corrected *p* = 0.004, following 20 min of immobilization, *t*(23) = 2.69, corrected *p* = 0.006, and an hour following the termination of immobilization, *t*(23) = 2.23, corrected *p* = 0.02.

### Experiment 3: Anxiety-Like Behavior and Replication of Glucocorticoid Abnormalities

#### Growth Rates

The analysis of growth rates revealed no significant difference between stressed females (*M* = 1.67 g/day, *SEM* = 0.08) and non-stressed females (*M* = 1.53 g/day, *SEM* = 0.07), *t*(38) = 1.37, *p* = 0.18.

#### EPM Behavior

**Figure 4:**
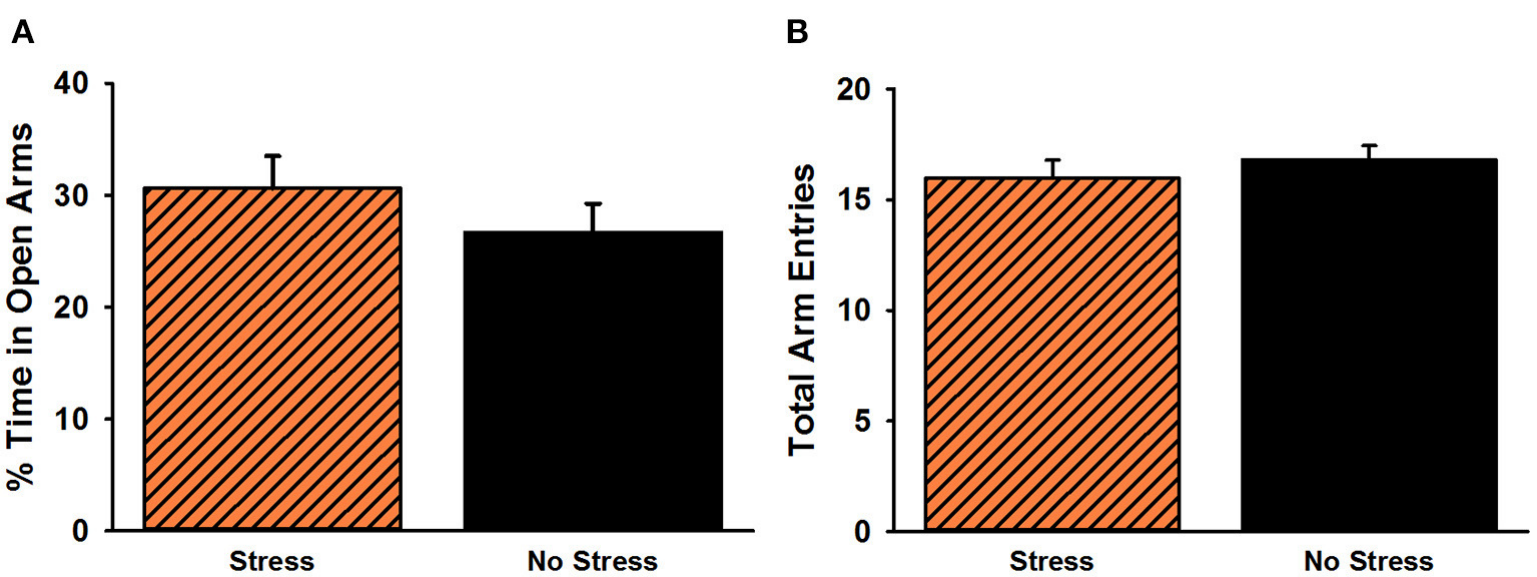
Percent time spent in the open arms **(A)** and total arm entries **(B)** on the elevated plus maze. No significant group differences were observed for either measure. Data are presented as means ± SEM.

The analysis of time spent in the open arms of the EPM revealed no significant difference between stressed females and non-stressed females, *t*(38) = 1.02, *p* = 0.31 (Figure 4). There was also no significant difference for overall arm entries on the EPM, *t*(38) = 0.80, *p* = 0.43.

#### Corticosterone Levels

**Figure 5:**
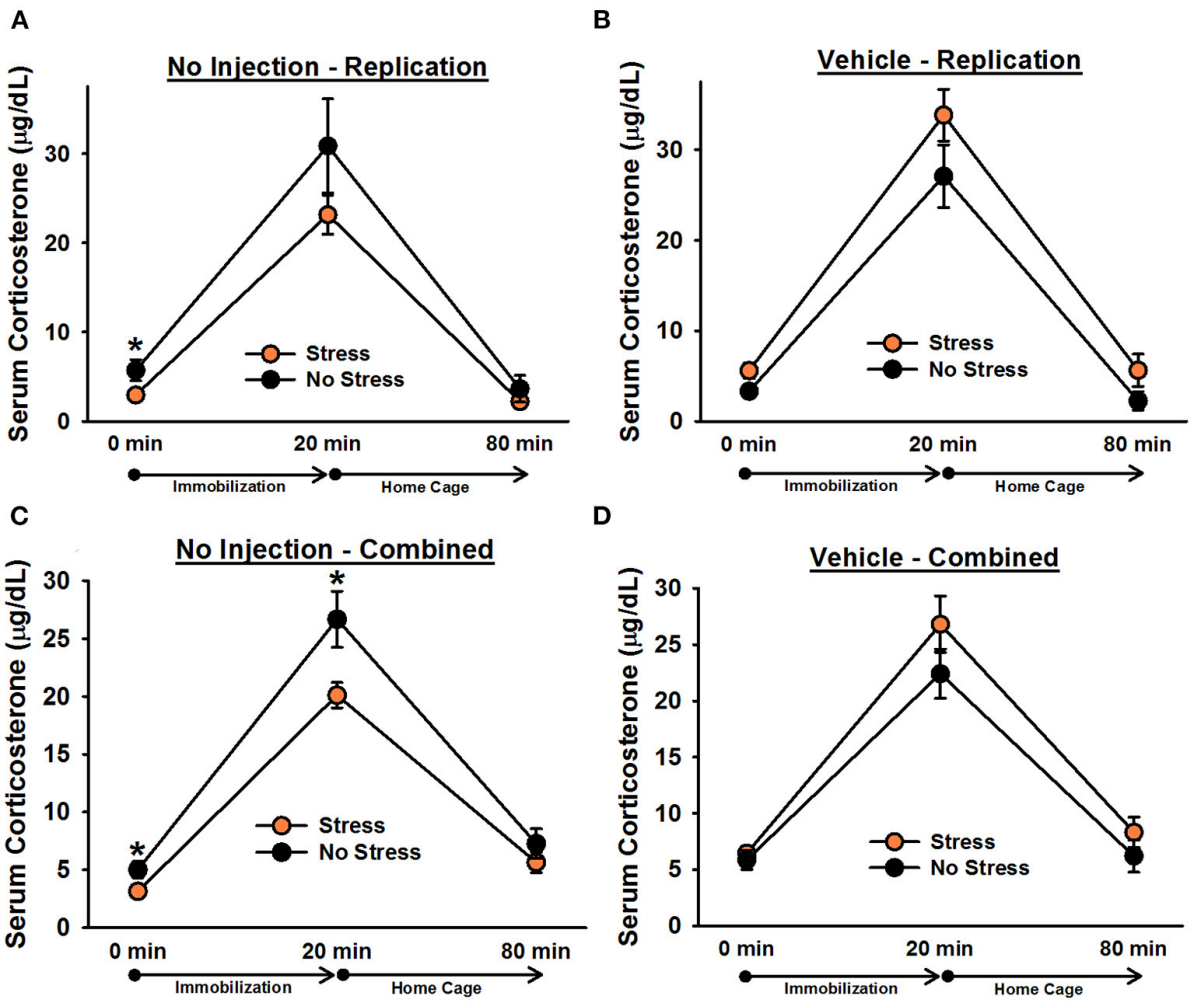
Serum corticosterone levels in non-injected **(A,C)** and vehicle-injected **(B,D)** female rats. In Experiment 3, non-injected, stressed females exhibited significantly lower overall corticosterone levels than non-stressed females **(A)**, replicating the effect observed in Experiment 1. Planned comparisons revealed that this effect was largely driven by a significant difference in corticosterone levels at baseline (0 min). Administration of vehicle 6 h prior to blood sampling reversed this effect and resulted in significantly greater overall serum corticosterone levels in stressed females **(B)**. Combining the data from non-injected and vehicle-injected female rats from all three experiments revealed that, under non-injected conditions **(C)**, stressed females exhibited significantly lower corticosterone levels than non-stressed females, particularly at the 0 and 20 min time points, and vehicle treatment eliminated any significant group differences **(D)**. Data are presented as means ± SEM. *p < 0.05.

There was a significant effect of time point, [*F*_(1.2, 70)_ = 170.60, *p* < 0.001]. *Post-hoc* tests indicated that corticosterone levels significantly increased following 20 min of immobilization, *t*(38) = 13.76, corrected *p* < 0.001, and then significantly decreased after rats spent an hour in their home cages, *t*(38) = 13.40, corrected *p* < 0.001 (Figure 5). There was also a significant Stress × Injection interaction, [*F*_(1, 35)_ = 7.98, *p* = 0.008]. *Post-hoc* tests showed that, consistent with the findings from Experiment 1, under non-injected conditions, stressed females tended to exhibit lower overall corticosterone levels than non-stressed females, *t*(18) = 1.66, corrected *p* = 0.055. In contrast, following vehicle injections, stressed females tended to exhibit *greater* overall corticosterone levels than non-stressed females, *t*(17) = 2.74, corrected *p* = 0.052. Moreover, non-injected stressed females displayed significantly lower overall corticosterone levels than vehicle-injected stressed females, *t*(18) = 4.61, corrected *p* = 0.009, while there was no significant difference between non-injected, non-stressed females and vehicle-injected, non-stressed females, *t*(17) = 0.95, corrected *p* = 0.227. Although the Stress × Injection × Time Point interaction was not significant, [*F*_(2, 70)_ = 1.57, *p* = 0.22], we performed Bonferroni-corrected comparisons to determine whether non-injected, stressed females exhibited significantly lower corticosterone levels than vehicle-injected, stressed females at each time point of blood sampling. These comparisons showed that non-injected, stressed females had significantly lower corticosterone levels than vehicle-injected, stressed females at baseline, *t*(18) = 2.57, corrected *p* = 0.036, and following 20 min of immobilization, *t*(18) = 2.97, corrected *p* = 0.043, but not an hour after the termination of immobilization, *t*(18) = 1.75, corrected *p* = 0.078. We also performed Bonferroni-corrected comparisons at each time point of blood sampling to examine corticosterone levels between stressed and non-stressed females that were injected with vehicle or were not injected. These comparisons revealed that, under non-injected conditions, stress females exhibited significantly lower corticosterone levels than non-stressed females at baseline, *t*(18) = 2.10, corrected *p* = 0.029.

To increase statistical power in our analyses, we combined the data from non-injected and vehicle-injected females from Experiments 1 and 2 with the data from Experiment 3. Analysis of the combined data revealed a significant Stress × Injection interaction, [*F*_(1, 84)_ = 11.78, *p* = 0.001]. *Post-hoc* tests indicated that, under non-injected conditions, stressed females exhibited significantly lower overall corticosterone levels than non-stressed females, *t*(50) = 2.88, corrected *p* = 0.002. In contrast, following vehicle injections, no significant differences were observed, *t*(34) = 2.19, corrected *p* = 0.068. Although the Stress × Injection × Time Point interaction was not significant, [*F*_(2, 168)_ = 2.34, *p* = 0.10], we performed Bonferroni-corrected comparisons to assess whether, under non-injected conditions, stressed and non-stressed females differed at any specific time points. These comparisons indicated that stressed females exhibited significantly lower corticosterone levels than non-stressed females at baseline, *t*(50) = 2.35, corrected *p* = 0.027, and following 20 min of immobilization, *t*(50) = 2.47, corrected *p* = 0.019.

## Discussion

Similar to males (Zoladz et al., 2012), female rats exposed to the predator-based psychosocial stress model of PTSD exhibited significantly lower serum corticosterone levels and a significantly greater suppression of corticosterone levels following the administration of dexamethasone. Interestingly, these group differences in corticosterone levels were observed despite an absence of anxiety-like behavior in stressed females. The observed alterations in HPA axis function are comparable to those observed extensively in people with PTSD (Lehrner et al., 2016). Thus, our findings suggest that this model may be useful in studying mechanisms that underlie trauma-induced changes in HPA axis function in females.

In Experiment 1, we assessed the impact of chronic stress on serum corticosterone levels in non-injected females. We did not observe a significant interaction between stress and blood sampling time point; instead, we observed a significant main effect of stress, suggesting that stressed females exhibited significantly lower overall corticosterone levels than non-stressed females. Nevertheless, planned comparisons indicated that this difference in overall corticosterone levels was largely driven by significantly lower corticosterone levels in stressed females following 20 min of immobilization. These findings differ from our previous findings in males in that stressed males selectively exhibited lower corticosterone levels at the 0 min time point (Zoladz et al., 2012, Figure 4). Our present findings are comparable to other preclinical work in female rodents. Specifically, Louvart and colleagues found that a single intense footshock, accompanied by subsequent situational reminders, led to significantly blunted corticosterone levels in female rats (Louvart et al., 2005, 2006). Interestingly, this effect was particularly noticeable when, similar to our methodology, the females were exposed to an acute stressor long after the single intense footshock.

In Experiment 2, we found that the administration of a vehicle solution 6 h prior to blood sampling was enough to obscure the group differences in corticosterone levels observed in Experiment 1. This is consistent with our previous work in stressed males (Zoladz et al., 2012) and emphasizes the sensitivity of HPA axis responses to acute, mild stress (intraperitoneal injection) on the day of blood sampling. Most importantly, stressed females exhibited significantly lower overall corticosterone levels than non-stressed females following the administration of 10 μg/kg, but not 25 μg/kg, dexamethasone. In previous work, we observed similar effects in stressed males (Zoladz et al., 2012), albeit those effects were observed for both 10 and 25 μg/kg doses of dexamethasone and were time-dependent (detected at the 80 and 20 min time points, respectively). While a few studies have reported similar effects in stressed males (e.g., Kohda et al., 2007; Zoladz et al., 2012; Pooley et al., 2018), we are aware of only one other study that has examined the dexamethasone suppression test in females exposed to intense stress. In that study (Pooley et al., 2018), the investigators reported that male, but not female, rats exhibited a greater suppression of corticosterone following dexamethasone treatment. It is possible that methodological differences could explain why, in contrast to our findings, Pooley et al. (2018) did not observe enhanced negative feedback of the HPA axis in stressed females, as they gave female rats a single cat exposure and performed blood sampling 1 week later. Ultimately, our observation of significantly lower corticosterone levels in stressed females following dexamethasone treatment suggests that these rats may have enhanced negative feedback regulation of the HPA axis, consistent with observations made in people with PTSD. Because dexamethasone poorly penetrates the blood-brain barrier (De Kloet, 1997; Meijer et al., 1998), this enhanced negative feedback may be particularly attributable to increased sensitivity of corticosteroid receptors in peripheral structures and central structures lying outside the blood-brain barrier (e.g., pituitary gland).

The purpose of Experiment 3 was to examine the impact of the predator-based psychosocial stress model of PTSD on anxiety-like behavior in females and to replicate the serum corticosterone differences observed in non-injected and vehicle-injected females in Experiments 1 and 2. In this follow-up experiment, we observed no significant group differences for anxiety-like behavior on the EPM. This finding is both consistent and inconsistent with our previous work. In 2015, we reported that female rats exposed to the predator-based psychosocial stress model of PTSD did not exhibit heightened anxiety on the EPM (Rorabaugh et al., 2015). We speculated that this lack of effect could be due to the impact of estrous-related hormone fluctuations on anxiety-like behavior in females. Thus, we subsequently controlled for the influence of estrous stage on the response of female rats to the stress paradigm. In such work, we found that stressed females, *independent* of estrous stage, spent significantly less time in the open arms of the EPM than non-stressed females (Zoladz et al., 2019, Experiment 1). In that study, we had a large amount of statistical power to detect such differences; we tested 77 stressed females and 87 non-stressed females to allow us to test rats from each of the four estrous stages. In other words, significant group differences in female behavior on the EPM may be attributable to small-to-moderate effect sizes and only be observed with larger sample sizes. Indeed, in the same manuscript, we subsequently failed to replicate (Zoladz et al., 2019, Experiment 3) the stress-induced increase in anxiety-like behavior on the EPM when testing only 20 stressed and 20 non-stressed females (similar to Experiment 3 here). It is also possible that anxiety-like behavior manifests differently in males and females as a result of the stress paradigm. Consistent with this speculation, previous work has shown that, relative to males, female rats exhibit less aversion to the open arms of the EPM at baseline and following chronic stress exposure (e.g., Johnston and File, 1991; Huynh et al., 2011). Moreover, previous research has suggested that the EPM is a more accurate measure of activity, as opposed to anxiety, in females (Fernandes et al., 1999). There is also work in humans reporting symptomatic differences in men and women with PTSD (Carmassi et al., 2014; Carragher et al., 2016; Murphy et al., 2019). In our own work, we have detected more consistent anxiogenic effects of the predator-based psychosocial stress model of PTSD on open field behavior in females (Zoladz et al., 2019), consistent with the notion that the EPM may be a better assessment of anxiety in males (Fernandes et al., 1999). However, additional research is necessary to verify this observation, and future work with this model will need to explore multiple measures of anxiety in females to determine which ones produce the most reliable findings.

Although we previously reported, in males, that a vehicle injection 6 h prior to blood sampling was enough to eliminate group differences in serum corticosterone levels, we wanted to replicate the comparable observation from females in Experiment 2 to verify that it was not attributable to a Type I error, inconsistent changes in serum corticosterone levels as a result of the stress paradigm, or a blunted response to chronic stress in vehicle-treated female rats. Consistent with our findings from Experiment 1, in Experiment 3, we found that non-injected, stressed females exhibited significantly lower overall corticosterone levels than non-injected, non-stressed females. This effect was largely driven by lower corticosterone levels in stressed females at baseline (0 min). Injecting rats with vehicle 6 h prior to blood sampling not only eliminated this effect, it reversed it; vehicle-injected, stressed females exhibited significantly greater overall corticosterone levels than vehicle-injected, non-stressed females. Vehicle-injected, stressed females also had significantly greater overall corticosterone levels than non-injected, stressed females. Although this finding differed from that of Experiment 2, it confirmed that vehicle injections could abolish the observation of significantly lower corticosterone levels in stressed rats. It is possible that, in Experiment 3, EPM testing on Day 32 had an impact on subsequent HPA axis function in rats, resulting in a greater sensitivity to vehicle injections and different results from those observed in Experiment 2. When we combined the data from non-injected and vehicle-injected animals across all three experiments, we observed that non-injected, stressed females exhibited significantly lower corticosterone levels than non-injected, non-stressed females, particularly at baseline and following acute immobilization. These effects were eliminated following the injection of vehicle.

One important limitation of our findings is that stress significantly reduced female growth rates in Experiment 1 but had no significant effect on growth rates in Experiments 2 and 3. We have observed similar variability in female growth rate data in prior work (Zoladz et al., 2019). One possibility for the inconsistency in these data is that different groups of females experience the acute cat exposures during different stages of the estrous cycle, which differentially affects body weight gain. Although we previously reported that the estrous cycle does not significantly impact behavioral outcomes of our model, we have not systematically examined its influence on how the model impacts body weight. Thus, the estrous cycle may interact with the stress in our model to differentially influence physiological measures that have yet to be thoroughly assessed.

## Conclusions

Most of our previous work with this model of PTSD has been performed in male rats. However, we recently examined the physiological and behavioral effects of the stress model in females. Similar to males, females exposed to the stress model exhibited long-lasting increases in anxiety-like behavior, as assessed on the elevated plus maze and in the open field (Zoladz et al., 2019) (however, see section discussion above). On the other hand, unlike males, stressed females did not exhibit an exaggerated startle response, nor did they display greater myocardial sensitivity to ischemic injury (Rorabaugh et al., 2015; Zoladz et al., 2019). Our present findings of PTSD-like glucocorticoid abnormalities in stressed females extend on this previous work and further validate the use of this model of examine trauma-induced changes in female physiology and behavior that may be helpful in understanding the mechanisms underlying PTSD.

## Data Availability Statement

The raw data supporting the conclusions of this article will be made available by the authors, without undue reservation.

## Ethics Statement

The animal study was reviewed and approved by Ohio Northern University Institutional Animal Care and Use Committee.

## Author Contributions

PZ designed the studies, collected all blood samples, wrote the initial draft of the manuscript, and completed the manuscript revision. CD, ES, CG, JD, KE, and CK performed the stress manipulations and behavioral testing, scored the behavioral data, and contributed to the initial draft of the manuscript. BR helped design the studies, performed all corticosterone assays, and contributed to the initial draft and revision of the manuscript. All authors contributed to the article and approved the submitted version.

## Conflict of Interest

The authors declare that the research was conducted in the absence of any commercial or financial relationships that could be construed as a potential conflict of interest.
